# An association between overexpression of DNA methyltransferase 3B4 and clear cell renal cell carcinoma

**DOI:** 10.18632/oncotarget.14966

**Published:** 2017-02-01

**Authors:** You Liu, Liantao Sun, Peter Fong, Jie Yang, Zhuxia Zhang, Shuihui Yin, Shuyuan Jiang, Xiaolei Liu, Hongge Ju, Lihua Huang, Jing Bai, Kerui Gong, Shaochun Yan, Chunyang Zhang, Guo Shao

**Affiliations:** ^1^ Biomedicine Research Center and Basic Medical College, Baotou Medical College, Inner Mongolia, PRC; ^2^ Department of Neurology, University of California San Francisco, San Francisco, CA, USA; ^3^ Department of Oral and Maxillofacial Surgery, University of California San Francsico, San Francisco, CA, USA; ^4^ Department of Neurology, First Affiliated Hospital of Baotou Medical College, Inner Mongolia, PRC; ^5^ Institute for Hypoxia Medicine, Capital Medical University, Beijing, PRC

**Keywords:** DNA methyltransferase 3B, clear cell renal cell carcinoma

## Abstract

It is well known that abnormal DNA methylations occur frequently in kidney cancer. However, it remains unclear exactly which types of DNA methyltransferases (DNMT) contribute to the pathologies of kidney cancers. In order to determine the functions of DNA methyltransferase in kidney tumorigenesis on the molecular level, we examined the mRNA expression levels of DNMT1, DNMT3A, DNMT3B, and DNMT3B variants in renal cell carcinoma tissue. Both mRNA and protein levels of DNMT3B4, a splice variant of DNMT3B, were increased in renal cell carcinoma tissue compared with adjacent control tissues. Additionally, Alu elements and long interspersed nuclear elements (LINE-1) were hypomethylated in renal cell carcinoma tissue. Meanwhile, methylation of the promoter for RASSF1A, a tumor suppressor gene, was moderately increased in renal cell carcinoma tissue, while RASSF1A expression was decreased. Thus, our data suggest that the overexpression of DNMT3B4 may play an important role in human kidney tumorigenesis through chromosomal instability and methylation of RASSF1A.

## INTRODUCTION

The worldwide incidence of kidney cancer is estimated at 337,860 new cases per year based on the International Agency for Research on Cancer's GLOBOCAN 2012 update, with an estimated 143,369 deaths annually [1]. Renal cell carcinoma (RCC) is the most common type of kidney cancer, and the five major subtypes of RCC are characterized by their histopathology. Approximately 75% of RCCs are defined as common or conventional renal cell carcinoma (clear cell renal cell carcinoma, ccRCC), while 10%, 5%, and 1% of RCCs are defined as papillary renal cell cancer, chromophobe renal cell cancer, and collecting-duct carcinoma, respectively. Approximately 3-5% of RCCs remain undefined [2].

Both genetic and epigenetic factors contribute to the regulation of biological pathways in the development and progression of ccRCC [3]. Thus, genomic and epigenomic changes that disrupt important biological functions and determine intra-tumor heterogeneity may provide a molecular basis for different RCC phenotypes, as well as a better understanding of the complex pathology of ccRCC [4]. Aberrant epigenetic alterations of DNA methylation in somatic cells are recognized as a hallmark of human carcinomas. Epigenetic DNA methylation is catalyzed by DNA methyltransferase (DNMT) and occurs at the 5-C position of cytosine nucleotides in CpG dinucleotides throughout genomic DNA. While hypermethylation of CpG dinucleotides near the promoter regions of some tumor suppressor genes induces transcriptional silencing of these genes, hypomethylation of CpG dinucleotides in other genomic regions have been associated with increased chromosomal instability [5].

Three DNMTs have been identified in directing mammalian genomic methylation patterns. While DNMT1 plays a critical role in maintaining DNA methylation patterns after DNA replication, DNMT3A and DNMT3B are responsible for *de novo* DNA methylation and modify unmethylated DNA during embryogenesis and germ cell development [6]. In cancer cells, DNMT expression becomes disregulated, and cells exhibit abnormal DNA methylation patterns that contribute to tumorigenesis. Robertson *et al*. demonstrated that in four different types of tumors, DNMT3B was significantly overexpressed, while DNMT1 and DNMT3A were only modestly overexpressed and with lower frequency [7]. Thus, the overexpression of DNMT3B, but not DNMT1 or DNMT3A, has been observed in several types of cancers, and suggests an important role for DNMT3B in tumorigenesis [8].

Some reports have indicated that in RCC, multiple tumor suppressor genes are simultaneously inactivated by promoter methylation [9, 10]. Additionally, the degree of methylation at LINE-1 and AluYb8 repeat sequences are also altered in RCC [11]. While changes in DMNT expression or function are likely an important part of the change in DNA methylation observed in RCC, it remains unclear which DNMTs contribute to this effect.

While DNMT3B has been shown to play an important role in tumorigenesis [8], the DNMT3B gene encodes nearly 40 known splice variants that are expressed in distinct tissues and are modulated in a disease-specific manner [12]. Thus, it remains unclear how each individual splice variants may contribute to DNMT3B function in normal and disease conditions. In this study, we investigated whether there are correlative relationships between the expression of various DNMT3B splice variants and ccRCC.

## RESULTS

### mRNA expression of DNMT1, DNMT3A, total DNMT3B, and DNMT3B variants in ccRCC tissue

**Figure 1:**
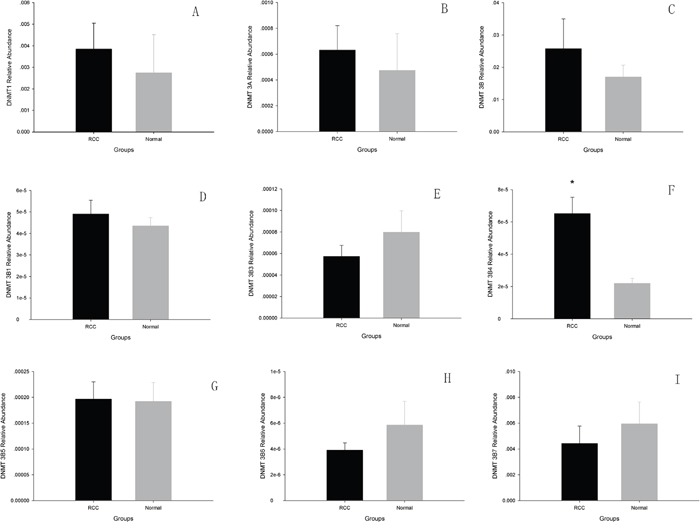
The analysis of DNMTs mRNA levels using Real-time PCR in ccRCC and adjacent normal tissues **A**. DNMT1; **B**. DNMT3A; **C**. total DNMT3B; **D**. DNMT3B1; **E**. DNMT3B3; **F**. DNMT3B4; **G**. DNMT3B5; **H**. DNMT3B6; **I**. DNMT3B7. (*p<0.05).

Fifteen pairs of tissue samples, which included ccRCC tissue and adjacent matched normal tissue from the patients, were analyzed by real-time PCR. We compared mRNA levels of DNMT1, DNMT3A, total DNMT3B, and DNMT3B variants in cancer tissue with those in matched adjacent normal tissue. We found no differences in the mRNA levels of DNMT1, DNMT3A, or total DNMT3B in ccRCC tissue and adjacent normal tissue (*p* > 0.05). However, DNMT3B4 mRNA expression was significantly greater in ccRCC tissue *(p* < 0.05), while those of other DNMT3B variants showed no difference in ccRCC tissue as compared with adjacent normal tissue (*p* >0.05) (Figure 1).

### The expression of DNMT3B4 protein is higher in ccRCC tissues than control

**Figure 2:**
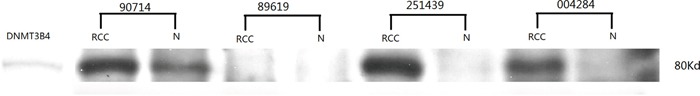
The analysis of DNMT3B4 expression using Western blot in ccRCC and adjacent normal tissues

Western blot analysis was used to determine DNMT3B4 expression in ccRCC tissue and adjacent normal tissue (Figure 2). DNMT3B4 protein was also loaded into each gel as a positive control. While DNMT3B4 protein was found in one-third (5/15) of the ccRCC tissues, it was absent in almost all of the normal tissues (14/15). Thus, consistent with our data from quantitative PCR, our results indicate greater expression of DNMT3B4 in ccRCC tissue (Figure 2).

### ccRCC tissue exhibited greater genomic unmethylation than control

**Figure 3:**
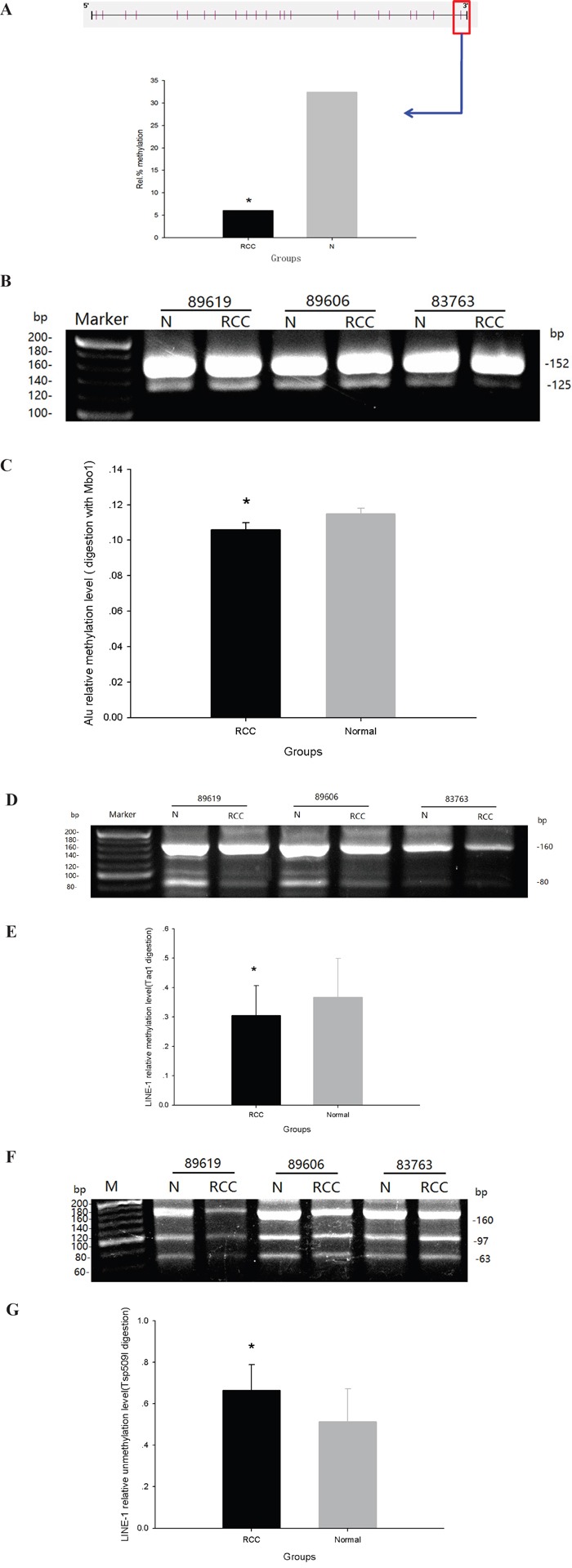
Global methylation of Sat2, Alu and LINE-1 in ccRCC and adjacent normal tissues **A**. Bisulfite-Modified DNA Sequencing (BMDS) analysis. **B**. Electrophoresis of COBRA products (Alu DNA sequence + Mbo1 digestion) in agarose gel. **C**. Rate of DNA methylation in Alu DNA sequence. **D**. Electrophoresis of COBRA products (LINE-1 DNA sequence + Taq1 digestion) in agarose gel. **E**. Rate of DNA methylation in LINE-1 DNA sequence. **F**. Electrophoresis of COBRA products (LINE-1 DNA sequence + TSP509I digestion) in agarose gel. G. Rate of DNA unmethylation in LINE-1 DNA sequence. (*p<0.05).

DNA methylation of satellite 2 repeats (394bp) in ccRCC and adjacent normal tissues was examined by Bisulfite-Modified DNA Sequencing. Satellite 2 repeat sequences contain 23 CpG dinucleotide sites, and no differences were found in the methylation of any of these sites between ccRCC tissue and adjacent normal tissues. However, methylation of the 23^rd^ nucleotide site was greatly diminished in ccRCC tissue (6%), as compared with normal tissue (32%) (Figure 3A). Additionally, methylation of Alu and LINE-1sequences in other genomic regions were decreased in ccRCC tissue. DNA methylation was calculated as the OD of digestion bands/digestion bands + indigestion bands. The relative methylation of Alu elements in ccRCC tissue and adjacent normal tissue, as detected by Mbo1 digestion, were 0.106 ± 0.04 and 0.115 ± 0.03, respectively (Figure 3B and 3C). The relative methylation of LINE-1 sequences in ccRCC tissue and adjacent normal tissue, as detected by Taq1 digestion, were 0.305 ± 0.102 and 0.367±0.132, respectively (Figure 3D and 3E). Finally, the relative unmethylation of LINE-1 sequences in ccRCC tissue and adjacent normal tissue, as detected by TSP509 I digestion, were 0.665 ± 0.123 and 0.513 ± 0.159, respectively (Figure 3E and 3F).

### ccRCC tissue exhibits a little hypermethylation of RASSF1A promoter coupled with decreased RASSF1A mRNA and protein expression

**Figure 4:**
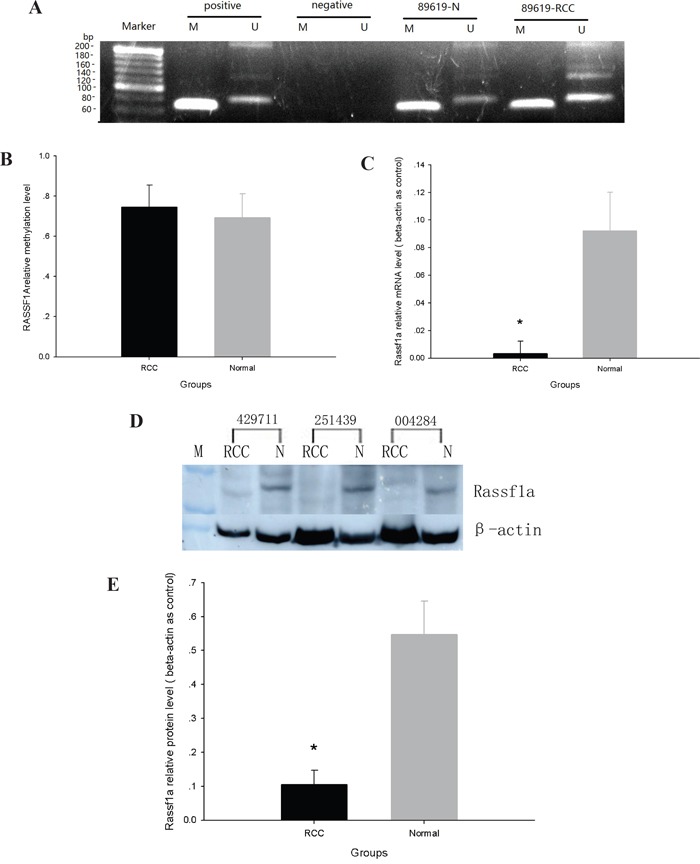
The expression and methylation of RASSF1A in ccRCC and adjacent normal tissues **A**. MS-PCR analysis of methylation of RASSF1A. **B**. Rate of DNA methylation in RASSF1A. **C**. Real-time PCR analysis of the ration of RASSF1A mRNA to β-actin mRNA. D. Western blot analysis of the expression of RASSF1A protein. **E**. Ratio of RASSF1A protein to beta-actin protein. (*p<0.05).

RASSF1A promoter methylation was determined by MSPCR. While RASSF1A promoter methylation was moderately elevated in ccRCC tissue (0.745 ± 0.11%) as compared with adjacent normal tissue (0.692 ± 0.12%), this finding was not statistically significant (*p*>0.05) (Figure 4A and 4B).

RASSF1A mRNA levels were measured by real-time PCR in ccRCC tissues and in adjacent normal tissues. The relative abundance of RASSF1A mRNA was calculated as the ratio of RASSF1A mRNA to β-actin mRNA and was found to be nearly three times less in ccRCC tissue (0.032 ± 0.009) than in normal tissue (0.0923 ± 0.028) (*p* < 0.05) (Figure 4C).

RASSF1A protein levels were detected in each cell clone by Western Blot analysis (Figure 4D) and were identified as approximately 39 kDa bands. The relative abundance of RASSF1A protein in each sample was calculated as the OD ratio of RASSF1A protein to β-actin. The relative abundance of RASSF1A protein in ccRCC tissue (0.15 ± 0.03) was found to be less than half of that found in adjacent normal tissue(0.37 ± 0.081) (*p* < 0.05) (Figure 4B and 4D).

## DISCUSSION

DNA methylation patterns in somatic cells are characteristically stable and are executed in a highly cell-specific manner. In certain types of cancer, these methylation patterns are aberrantly altered by genetic and epigenetic factors [13, 14] and even exhibit distinct patterns of methylation that enable certain cancers to be described by their DNA methylation signatures [15–18]. Aberrant hypermethylation of DNA may occur near the promoter regions of tumor suppressor genes and inhibit the transcription and activation of these genes in tumorigenesis. Additionally, abnormal hypomethylation may also occur at various DNA repeat sequences throughout the genome, resulting in chromosomal instability and rearrangement [19]. In RCC, both of these effects have been reported to occur [10, 11]. A report displayed the evidence of epigenetic silenced at least 5% of 289 genes in ccRCC using DNA methylation arrays [3]. Higher levels of LINE-1 methylation in blood DNA have also been associated with increased risk of developing RCC in populations [20]. To date, it has not been identified exactly which DNMTs contribute to these changes in DNA methylation patterns observed in RCC.

One promising avenue of investigation in the study of abnormal DNA methylation which occurs in RCC is the family of DNMTs which catalyze the transfer of methyl groups on to DNA. mRNA expression of DNMT1, DNMT3A, DNMT3B, and six different isoforms of DNMT3B were examined by real-time PCR, with only DNMT3B4 exhibiting significantly different expression levels between cancer tissues and adjacent normal tissues. DNMT3B4 protein was observed to be overexpressed in one-third of the ccRCC patients included in this study, suggesting a potential relationship between increased DNMT3B4 expression and ccRCC development.

While it may be reasonable to presume that the increased expression or functional activity of DNMT results in greater aberrant DNA methylation patterns leading to tumorigenes is in cancer cells, Ostler *et al*. concluded that DNMT expression does not correlate with the aberrant DNA methylation found in cancer cells [21]. However, it should be noted that it is DNMT3B, but not DNMT1 or DNMT3A, that has been implicated in aberrant DNA methylation in tumorigenesis for several types of cancer [7, 22, 23]. Thus, the discrepancy that was observed by Ostler et al. could be explained by the fact that different results may be obtained when assaying different DNMT isoforms [22, 23]. Indeed, this is also consistent with our results demonstrating that ccRCC tissue exhibited significantly greater mRNA and protein levels of DMNT3B4, but not DNMT1, or DNMT3A, than normal tissue. While DNMT3B3 is the dominant DNMT3B isoform in human somatic cells and is ubiquitously expressed in all normal tissues [24], nearly 40 isoforms of DNMT3B exist in human somatic cells. Thus, it is reasonable to expect that a change in the concentration of one isoform, which is in relatively low abundance, will not produce a large change in overall DNMT3B levels. Indeed, our results from real-time PCR analysis, which indicate that the relative abundance of DNMT3B4 mRNA to total DNMT3B mRNA is 1 to 1000, supports the hypothesis that while there may be no difference in DNMT3B mRNA expression between ccRCC tissue and normal tissue, DNMT3B4 overexpression plays an important role in tumorigenesis is in ccRCC.

Consistent with our results, overexpression of DNMT3B4 has also been correlated with DNA hypomethylation of pericentromeric satellite regions in other cancers such as hepatocellular carcinoma [22, 23]. In this study, we observed DNA hypomethylation of Alu and LINE-1 sequences in ccRCC tissue. Additionally, it is also interesting to note that other DNMT3B splice variants may also preferentially interact with specific genomic sequences [25]. DNMT3B splice variants can be divided into two groups that are defined by whether or not these variants exhibit DNA methyltransferase activity [23]. It is likely that DNMT3B4 lacks DNA methyltransferase activity, since it lacks the conservative sequence motifs IX and X, which are responsible for enzymatic activity, and is believed to act in negative regulation of DNA methylation [23]. Gordon *et al*. demonstrated through an *in vivo* episomal assay that DNMT3B4 broadly inhibits DNMT3 function. Thus, it is possible that DNMT3B4 overexpression may induce DNA demethylation of satellite 2 in ccRCC tissue.

DNMT3B4 overexpression may also induce DNA hypermethylation of antioncogenes in ccRCC. In RCC, hypermethylation of the promoter region of the tumor suppressor gene Ras association domain family 1A (RASSF1A) results in inactivation of this gene [26]. Although there was not significant difference between ccRCC and adjacent normal tissues, RASSF1A's promoter DNA methylation was found to be slightly increased. It may be due to the fact that the size of samples is too small.

In the present study, we found that RASSF1A expression was decreased in ccRCC tissue. Similar to DNMT3B4, DNMT3B7 is a truncated splice variant that is found in numerous types of tumors, lacks the conserved sequence motifs IX and X, and likely also lacks DNA methyltransferase activity [23, 27]. DNMT3B7 induces not only changes in DNA methylation patterns but also chromosomal rearrangements [21]. The overexpression of DNMT3B4 and DNMT3B7 in HEK 293 cells line may critically contribute to the altered DNA methylation and corresponding changes in gene expression that are observed in several types of cancer [21, 23]. Ostler*et al*. suggested that the abnormal DNA methylation patterns which are present in nearly all cancer cells may be regulated by enzymatically inactive DNMT3B proteins [21].

In conclusions, we measured the expression of DNMT1, DNMT3A, total DNMT3B, and six isoforms of DNMT3B that are found in normal tissue and in ccRCC tissue. We found that only DNMT3B4 was overexpressed in ccRCC tissue. Our data suggests that DNMT3B4 overexpression may contribute an important role in both the global and local genomic changes that occur in ccRCC.

## MATERIALS AND METHODS

### Tissue samples

Tissue samples consisted of 15 cancer tissues and 15 matched adjacent tissues from ccRCC patients who had undergone nephrectomy in 2012-2014 at the First Affiliated Hospital of Baotou Medical College (Table 1). This study acquired consent from all the patients and was approved by the Baotou Medical College ethics committee.

**Table 1:** The information of ccRCC patients

Number	Gender	Age	Inpatient number	Position
**1**	F	67	89619	right
**2**	F	74	89606	left
**3**	M	55	83763	right
**4**	M	47	90714	left
**5**	F	72	90731	righ
**6**	F	59	90583	left
**7**	F	75	90323	right
**8**	F	56	90108	right
**9**	M	68	004027	left
**10**	M	43	003344	left
**11**	M	45	002284	left
**12**	F	62	003777	right
**13**	M	51	429711	right
**14**	M	38	251439	left
**15**	F	61	004284	left

### Real-time PCR

RNA was collected from the tissue samples with an RNAeasy mini kit (Qiagen, Valencia, CA, USA), and cDNA was synthesized with Invitorgen Superscript III (Invitrogen, Carlsbad, CA, USA). The primers that were used for real-time PCR were as follows:

**Table:** 

DNMT1	F:AACCTTCACCTAGCCCCAG	R:CTCATCCGATTTGGCTCTTTCA
DNMT3A	F:GACAAGAATGCCACCAAAGC	R:CCATCTCCGAACCACATGAC
DNMT3B	F:AGGGAAGACTCGATCCTCGTC	R:CGTCTCCGAACCACATGAC
DNMT3B1	F:AGAATCAAGGAAATACGAGAACAA	R:ATCTTCATCCCCTCGGTCTTT
DNMT3B3	F:CCGGGATGAACAGGATCTTT	R:AGTAGTCCTTCAGAGGGGCG
DNMT3B4	F:CGGTTCCTGGAGTGTAATCC	R:GGTTATTGTCTGTACTTTCTTTAACTGTT
DNMT3B5	F:AATACAATAGGATAGCCAAGGATCT	R:TTCAGAGGGGCGAAGAGG
DNMT3B6	F:CCAAGCTTGGAAAGCATGAA	R:CCGTTGACGAGGATCGAGT
DNMT3B7	F:CAGTCTAATTACCTTTCACAGAGAACA	R: GTCTTGAGGCGCTTGGGT
RASSF1A	F:AGGACGGTTCTTACACAGGCT	R:TGGGCAGGTAAAAGGAAGTGC
Beta-actin	F:CATGTACGTTGCTATCCAGGC	R: CTCCTTAATGTCACGCACGAT

All PCR reactions were conducted in an ABI-7900 real-time PCR machine (Applied Biosystems, Singapore) with the following protocol: initial denaturation at 95°C for 10 min, 40 cycles at 95°C for 30 sec, 40 cycles at 60°C for 60 sec, and a final extension at 60°C for 2 min in a 50 μl reaction mixture containing 2 μl of each cDNA, 0.2 μM of each primer, and 25 μl 2x real-time master mix.

### Western blot

Protein from each tissue sample was extracted with RIPA buffer (Beyotime Institute of Biotechnology, China). Protein concentrations were determined with the bicinchoninic acid (BCA) method. Cell lysate (40 μg) was separated by SDS/PAGE (12%) at 30 mA for 2.5 h and then blotted onto a nitrocellulose membrane (Roche Diagnostics, Indianapolis, IN, USA). The membrane was then incubated for 1h in blocking buffer (Tris-buffered saline containing 10% skimmed milk powder) at room temperature. Next, the membrane was incubated for 16 h at 4°C with a rabbit anti-DNMT3B polyclonal antibody (Novus Biologicals, Littleton, CO, USA), a rabbit anti-RASSF1A polyclonal antibody (Novus Biologicals, Littleton, CO, USA), and a mouse anti-β-actin polyclonal antibody (Sigma, St. Louis, Mo.), followed by incubation with secondary antibodies (Tiangen biological, Beijing, China) for 1h at room temperature. After each antibody incubation, membranes were washed three times with Tris-buffered saline containing 0.05% tween20. Protein signals were detected by an electrochemiluminescence detection system (Pierce Biotechnology, Rockford, IL., USA), in which membranes were exposed to the detection solution for 5 min.

### DNA methylation of satellite DNA, Alu elements, and long interspersed nucleotide elements (LINE-1)

DNA from cancer tissues and adjacent normal tissues was extracted using a commercial kit (Qiagen DNA extraction kit, Qiagen Inc., Valencia, CA, USA), and bisulphite treatment was conducted withan EZ DNA Methylation kit (ZYMO Research, Irvine, CA). Following bisulphite treatment, modified DNA was amplified by PCR with the following satellite 2 primers: 5′-GAATTATTGAATAGAATTGAATGG-3′ and 5′-TAAATAATAACTCCTTTCATTT-3′. The resulting PCR products were gel-purified and then cloned into PCR2.1 vectors (Invitrogen, Carlsbad, CA, USA). A minimum of 10 clones were sequenced. We then performed Mann-Whitney *U*-test for analysis of DNA methylation of bisulfite genomic sequences. A similar assay, COBRA (combined bisulfite restriction analysis), was used to examine the methylation of Alu and LINE-1 sequences. Following bisulphite treatment, modified DNA was amplified by PCR with Alu and LINE-1 primers:

Alu-F:5′-GATCTTTTTATTAAAAATATAAAAATTAGT-3′; Alu-R:5′-GATCCCAAACTAAAATACAATAA-3′; LINE-F:5′-CCGTAAGGGGTTAGGGAGTTTTT-3′; LINE-R:5′-RTAAAACCCTCCRAACCAAATATAAA-3′.

The PCR products of Alu were digested with 10 U of MboI, while those of LINE-1 were digested with 10 U of either TaqI or TSP509I. The digested PCR products were then separated by 3% agarose gel electrophoresis.

### RASSF1A methylation-specific PCR (MS-PCR)

RASSF1A methylation was determined by sodium bisulfite treatment of DNA as described above, followed by MS-PCR. The MS-PCR primers and the experimental conditions were identical to those reported by Greenspan *et al*.[28]. Nested PCR was performed to increase reaction sensitivity. The primers used for the first iteration of PCR were: 5′-GTTTAGTTTGGATTTTGGGGGAG-3′ (sense) and 5′-CCCRCAACTCAATAAACTCAAACT-3′ (antisense). The resulting 144 bp fragment was used as a template for the MSP reaction. A 1:100 dilutions of the first PCR reaction were prepared, and the methylated and unmethylated products were amplified using the same reaction mixture as above. A 76 bp methylated product was amplified using the following primers: 5′-GGG TTC GTT TTG TGG TTT CGT TC-3′ (sense) and 5′-TAA CCCGAT TAA ACC CGT ACT TCG-3′ (antisense). An 81 bp unmethylated product was amplified using the following primers: 5′-GGG GTT TGT TTT GTG GTT TTG TTT-3′ (sense) and 5′-AAC ATA ACC CAA TTA AAC CCA TAC TTC A-3′ (antisense). Each PCR product (30 μl) was loaded onto a 2% agarose gel, stained with ethidium bromide and visualized under UV illumination.

### Quantification and statistical analysis

The optical density (OD) of bands produced by Western Blot and PCR experiments were obtained with a Gel-Doc system and analyzed with SigmaGel (Jandel Scientific, San Rafael, Calif., USA). All western blot data were normalized to β-actin and are presented as relative abundances. The threshold cycle (CT) value for target genes were normalized to those of β-actin reference genes and were calculated as ΔCT = CT_target_ − CT_β-actin_. Relative mRNA concentrations for each target gene were expressed as F = 2^ΔCT^. All data are expressed as mean ± SD (standard deviation). Statistical analysis was performed with an analysis of variance (ANOVA) model for intra-group comparisons and the Tukey HSD test for comparisons made between different groups. All statistical analyses were conducted with SPSS Version 10.0 (SPSS Inc., Chicago, IL., USA), and only those results with p < 0.05 were considered to be statistically significant.

We thank Mrs. Jean Danforth for her language-editing assistance.

## SUPPLEMENTARY MATERIALS FIGURES AND TABLES


