# Supplementary material for: An association between overexpression of DNA methyltransferase 3B4 and clear cell renal cell carcinoma
Source: Oncotarget. 2017 Feb 1;8(12):19712–22. doi: 10.18632/oncotarget.14966 (PMC5386716; doi:10.18632/oncotarget.14966)
Supplement: Supplementary file 1 [file oncotarget-08-19712-s001.pdf]

An association between overexpression of DNA methyltransferase 3B4 and clear cell renal cell carcinoma

SUPPLEMENTARY FIGURE

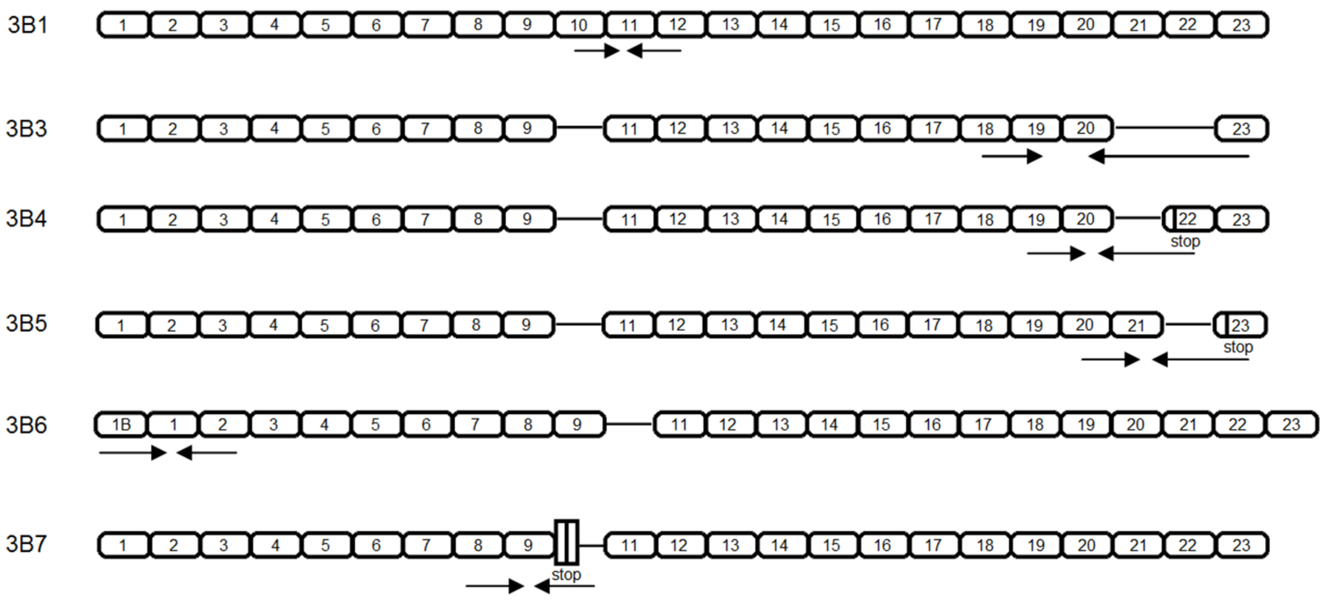

Supplementary Figure 1: Schematic showing the structure of six kinds of the human DNMT3B splice variants. Arrows showing the location of the primers.
